# (7*R*,8*R*,8a*S*)-8-Hydr­oxy-7-phenyl­per­hydro­indolizin-3-one

**DOI:** 10.1107/S160053680901085X

**Published:** 2009-03-28

**Authors:** Ľubomír Švorc, Viktor Vrábel, Jozefína Žúžiová, Mária Bobošíková, Jozef Kožíšek

**Affiliations:** a Institute of Analytical Chemistry, Faculty of Chemical and Food Technology, Slovak Technical University, Radlinského 9, SK-812 37 Bratislava, Slovak Republic 81237; b Institute of Organic Chemistry, Catalysis and Petrochemistry, Faculty of Chemical and Food Technology, Slovak Technical University, Radlinského 9, SK-812 37 Bratislava, Slovak Republic 81237; c Institute of Physical Chemistry and Chemical Physics, Faculty of Chemical and Food Technology, Slovak Technical University, Radlinského 9, Bratislava, Slovak Republic 81237

## Abstract

The absolute configuration of the title compound, C_14_H_17_NO_2_, was assigned from the synthesis. There are two mol­ecules in the asymmetric unit. Their geometries are very similar and corresponding bond lengths are almost identical [mean deviation for all non-H atoms = 0.015 (2) Å]. The six-membered ring of the indolizine system adopts a chair conformation. In the crystal structure, mol­ecules form chains parallel to the *a* axis *via* inter­molecular O—H⋯O hydrogen bonds, which help to stabilize the crystal structure.

## Related literature

Polyhydroxy­lated indolizidine alkaloids are excellent inhibitors of biologically important pathways, see: Melo *et al.* (2006 ▶); Michael (2003 ▶); Lillelund *et al.* (2002 ▶); Gerber-Lemaire & Juillerat-Jeanneret (2006 ▶); Butters (2002 ▶); Compain & Martin (2001 ▶); Shi *et al.* (2008 ▶); Fujita *et al.* (2004 ▶). For indolizines as anti­mycobacterial agents against mycobacterial tuberculosis, see: Gundersen *et al.* (2003 ▶). For the biological activity of indolizine derivatives, see: Teklu *et al.* (2005 ▶); Foster *et al.* (1995 ▶). For their pharmacological applications, see: Couture *et al.* (2000 ▶); Jorgensen *et al.* (2000 ▶). For puckering parameters, see: Cremer & Pople (1975 ▶). For conjugation of the lone-pair electrons in simple amides, see: Brown & Corbridge (1954 ▶); Pedersen (1967 ▶). For bond lengths and angles in related structures, see: Vrábel *et al.* (2004 ▶); Švorc *et al.* (2008 ▶). For the synthesis, see: Šafář *et al.* (2009 ▶).
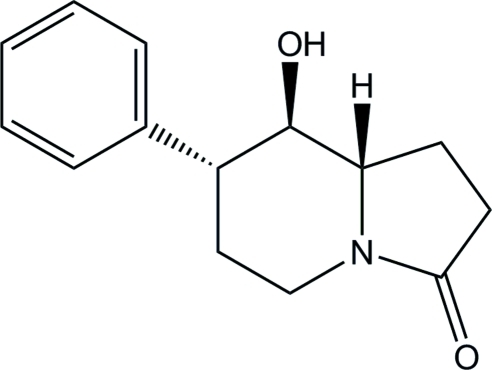


## Experimental

### 

#### Crystal data

C_14_H_17_NO_2_
*M* *_r_* = 231.29
Orthorhombic, 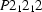

*a* = 25.3592 (4) Å
*b* = 16.1467 (2) Å
*c* = 6.0086 (1) Å
*V* = 2460.33 (6) Å^3^
*Z* = 8
Mo *K*α radiation
μ = 0.08 mm^−1^
*T* = 298 K
0.33 × 0.26 × 0.15 mm

#### Data collection

Oxford Diffraction Gemini R CCD diffractometer
Absorption correction: analytical (Clark & Reid, 1995 ▶) *T*_min_ = 0.965, *T*_max_ = 0.988
60218 measured reflections
3791 independent reflections
1856 reflections with *I* > 2σ(*I*)
*R*_int_ = 0.035

#### Refinement

*R*[*F* ^2^ > 2σ(*F* ^2^)] = 0.033
*wR*(*F* ^2^) = 0.097
*S* = 0.98
3791 reflections
311 parameters
H-atom parameters constrained
Δρ_max_ = 0.12 e Å^−3^
Δρ_min_ = −0.11 e Å^−3^

### 

Data collection: *CrysAlis CCD* (Oxford Diffraction, 2006 ▶); cell refinement: *CrysAlis RED* (Oxford Diffraction, 2006 ▶); data reduction: *CrysAlis RED*; program(s) used to solve structure: *SHELXS97* (Sheldrick, 2008 ▶); program(s) used to refine structure: *SHELXL97* (Sheldrick, 2008 ▶); molecular graphics: *DIAMOND* (Brandenburg, 2001 ▶); software used to prepare material for publication: *enCIFer* (Allen *et al.*, 2004 ▶).

## Supplementary Material

Crystal structure: contains datablocks I, global. DOI: 10.1107/S160053680901085X/fj2203sup1.cif

Structure factors: contains datablocks I. DOI: 10.1107/S160053680901085X/fj2203Isup2.hkl

Additional supplementary materials:  crystallographic information; 3D view; checkCIF report

## Figures and Tables

**Table 1:** Hydrogen-bond geometry (Å, °)

*D*—H⋯*A*	*D*—H	H⋯*A*	*D*⋯*A*	*D*—H⋯*A*
O4—H4⋯O1^i^	0.82	1.92	2.7366 (19)	175
O2—H2⋯O3^ii^	0.82	1.88	2.6963 (18)	179

Symmetry codes: (i) 
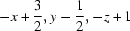
; (ii) 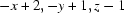.

**Fig. 1.**
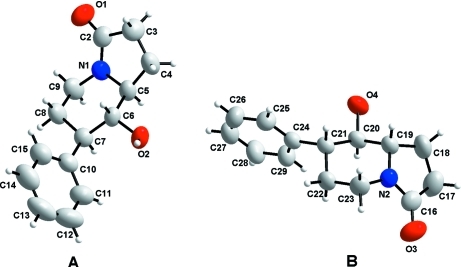
Molecular structure of (I) with the atomic numbering scheme. Displacement ellipsoids are drawn at the 50% probability level (Brandenburg, 2001).

**Fig. 2.**
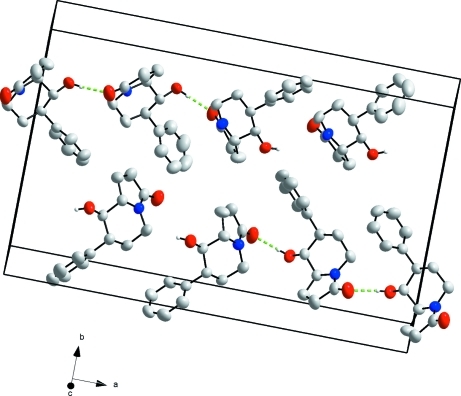
A packing of the molecule of (I), viewed along the a axis.

## supplementary crystallographic information

### Comment

The synthesis of biologically active indolizine derivatives continues to attract the attention of organic chemists, because of their wide spectrum of biological activity. Indolizines are natural structures, which are remarkable in its diversity and efficacy. For example, polyhydroxylated indolizidine alkaloids represented by the so popular castanospermine and swainsonine are well known for their ability to function as excellent inhibitors of biologically important pathways. These include the binding and processing of glycoproteins, potent glycosidase inhibitory activities (Melo *et al.*, 2006; Michael, 2003; Lillelund *et al.*, 2002), activity against AIDS virus HIV and some carcinogenic cells as well as against other important pathologies (Gerber-Lemaire & Juillerat-Jeanneret, 2006; Butters, 2002; Compain & Martin, 2001). More importantly, some hybrids of these structures have shown in numerous cases an increase of glycosidase activities as demonstrated by the Pearson's group and others (Shi *et al.*, 2008; Fujita *et al.*, 2004). Indolizines have also been tested as antimycobacterial agents against mycobacterial tuberculosis (Gundersen, *et al.*, 2003). Many studies demonstrated that indolizine derivatives show biological activity such as antioxidative (Teklu *et al.*, 2005) and antiherpes (Foster *et al.*, 1995). The other well known pharmacological applications associated with this ring compounds are well documented in the literature (Couture *et al.*, 2000; Jorgensen *et al.*, 2000).

Due to the diverse properties of indolizine derivatives, the structure of the title compound, (I), has been determined as part of our study of the conformational changes caused by different substituents at various positions on the indolizine ring system. We report here the synthesis, molecular and crystal structure. The absolute configuration was established by synthesis and is depicted in the scheme and figure. The asymmetric unit of title compound contains two crystallographic independent molecules as shown in Fig. 1. The expected stereochemistry of atoms C5, C6 and C7 (C19, C20 and C21 for molecule B) was confirmed as *S*, *R* and *R*, respectively. The corresponding bond lengths and angles in the independent molecules agree with each other and are almost identical (mean deviation for all non-H atoms 0.015 (2) Å). The central six-membered N-heterocyclic ring is not planar and adopts a chair conformation (Cremer & Pople, 1975). A calculation of least-squares planes shows that this ring is puckered in such a manner that the four atoms C5, C6, C8 and C9 (C19, C20, C22 and C23 for molecule B) are coplanar to within 0.012 (2)Å [0.014 (1) Å], while atoms N1 (N2) and C7 (C21) are displaced from this plane on opposite sides, with out-of-plane displacements of -0.573 (2) and 0.639 (2)Å [-0.573 (1) and 0.664 (2)Å for molecule B], respectively. The phenyl ring attached to the indolizine ring system is planar (mean deviation is 0.009 (2)Å for molecule A and 0.011 (2)Å for molecule B). As shown in Table of geometric parameters, the N1—C5 (N2—C19) and N1—C9 (N2—C23) bonds are approximately equivalent and both are much longer than the N1—C2 (N2—C16) bond. Moreover, the N1 (N2) atom is *sp*^2^ hybridized, as evidenced by the sum of the valence angles around it [359.8 (2)° for molecule A and 358.4 (2)° for molecule B]. These data are consistent with conjugation of the lone-pair electrons on N1 (N2) with the adjacent carbonyl and agree with literature values for simple amides (Brown & Corbridge, 1954; Pedersen, 1967). The bond length of the carbonyl group C2=O1 (C16=O3) is 1.228 (2)Å [1.229 (2) Å], respectively, is somewhat longer than typical carbonyl bonds. This may be due to the fact that atoms O1 and O3 participate as acceptors in intermolecular hydrogen bonds with atoms O4 and O2 as donators. These intermolecular O—H···O hydrogen bonds link the molecules of (I) into extended chains, which run parallel to the *a* axis (Fig. 2) and help to stabilize the crystal structure of the compound. The bond lengths and angles in the indolizine ring system are in good agreement with values from the literature (Vrábel *et al.*, 2004, Švorc *et al.*, 2008).

### Experimental

The title compound (7*R*,8*R*,8aS)-8-hydroxy-7-phenylhexahydroindolizin-3(5*H*)-one was prepared according literature procedures of Šafář *et al.* (2009).

### Refinement

All H atoms were placed in geometrically idealized positions and constrained to ride on their parent atoms, with C—H distances in the range 0.93 - 0.98Å and O—H distance 0.85Å and *U*_iso_ set at 1.2*U*_eq_ of the parent atom. The absolute configuration could not be reliably determined for this compound using Mo radiation, and has been assigned according to the synthesis. Friedel pairs have been merged.

### Crystal data

**Table:**

C_14_H_17_NO_2_	*F*(000) = 992
*M**_r_* = 231.29	*D*_x_ = 1.249 Mg m^−^^3^
Orthorhombic, *P*2_1_2_1_2	Mo *K*α radiation, λ = 0.71073 Å
Hall symbol: P 2 2ab	Cell parameters from 19073 reflections
*a* = 25.3592 (4) Å	θ = 3.0–29.5°
*b* = 16.1467 (2) Å	µ = 0.08 mm^−^^1^
*c* = 6.0086 (1) Å	*T* = 298 K
*V* = 2460.33 (6) Å^3^	Block, white
*Z* = 8	0.33 × 0.26 × 0.15 mm

### Data collection

**Table:**

Oxford Diffraction Gemini R CCD diffractometer	3791 independent reflections
Radiation source: fine-focus sealed tube	1856 reflections with *I* > 2σ(*I*)
graphite	*R*_int_ = 0.035
Detector resolution: 10.4340 pixels mm^-1^	θ_max_ = 29.6°, θ_min_ = 3.0°
Rotation method data acquisition using ω and φ scans	*h* = −34→34
Absorption correction: analytical (Clark & Reid, 1995)	*k* = −22→22
*T*_min_ = 0.965, *T*_max_ = 0.988	*l* = −8→8
60218 measured reflections	

### Refinement

**Table:**

Refinement on *F*^2^	Secondary atom site location: difference Fourier map
Least-squares matrix: full	Hydrogen site location: inferred from neighbouring sites
*R*[*F*^2^ > 2σ(*F*^2^)] = 0.033	H-atom parameters constrained
*wR*(*F*^2^) = 0.097	*w* = 1/[σ^2^(*F*_o_^2^) + (0.0552*P*)^2^] where *P* = (*F*_o_^2^ + 2*F*_c_^2^)/3
*S* = 0.98	(Δ/σ)_max_ = 0.001
3791 reflections	Δρ_max_ = 0.12 e Å^−^^3^
311 parameters	Δρ_min_ = −0.10 e Å^−^^3^
0 restraints	Extinction correction: *SHELXL97* (Sheldrick, 2008), Fc^*^=kFc[1+0.001xFc^2^λ^3^/sin(2θ)]^-1/4^
Primary atom site location: structure-invariant direct methods	Extinction coefficient: 0.0058 (10)

### Special details

**Table:**

Experimental. face-indexed (Oxford Diffraction, 2006)
Geometry. All e.s.d.'s (except the e.s.d. in the dihedral angle between two l.s. planes) are estimated using the full covariance matrix. The cell e.s.d.'s are taken into account individually in the estimation of e.s.d.'s in distances, angles and torsion angles; correlations between e.s.d.'s in cell parameters are only used when they are defined by crystal symmetry. An approximate (isotropic) treatment of cell e.s.d.'s is used for estimating e.s.d.'s involving l.s. planes.
Refinement. Refinement of *F*^2^ against ALL reflections. The weighted *R*-factor *wR* and goodness of fit *S* are based on *F*^2^, conventional *R*-factors *R* are based on *F*, with *F* set to zero for negative *F*^2^. The threshold expression of *F*^2^ > σ(*F*^2^) is used only for calculating *R*-factors(gt) *etc*. and is not relevant to the choice of reflections for refinement. *R*-factors based on *F*^2^ are statistically about twice as large as those based on *F*, and *R*- factors based on ALL data will be even larger.

### Fractional atomic coordinates and isotropic or equivalent isotropic displacement parameters (Å^2^)

**Table:**

	*x*	*y*	*z*	*U*_iso_*/*U*_eq_	
C2	0.70322 (7)	0.68436 (12)	0.1107 (4)	0.0598 (5)	
C3	0.74476 (8)	0.61874 (15)	0.0828 (4)	0.0771 (6)	
H3A	0.7286	0.5657	0.0504	0.093*	
H3B	0.7683	0.6330	−0.0384	0.093*	
C4	0.77424 (8)	0.61487 (12)	0.2968 (4)	0.0741 (6)	
H4A	0.8116	0.6233	0.2714	0.089*	
H4B	0.7693	0.5614	0.3673	0.089*	
C5	0.75196 (6)	0.68383 (11)	0.4430 (4)	0.0578 (5)	
H5	0.7375	0.6596	0.5796	0.069*	
C6	0.79146 (6)	0.75095 (10)	0.5042 (4)	0.0518 (5)	
H6	0.8110	0.7671	0.3704	0.062*	
C7	0.76420 (7)	0.82764 (11)	0.6015 (4)	0.0574 (5)	
H7	0.7480	0.8109	0.7425	0.069*	
C8	0.72007 (7)	0.85684 (11)	0.4495 (4)	0.0696 (6)	
H8A	0.7351	0.8763	0.3105	0.083*	
H8B	0.7020	0.9031	0.5189	0.083*	
C9	0.68041 (7)	0.78853 (12)	0.4007 (4)	0.0741 (6)	
H9A	0.6622	0.7728	0.5361	0.089*	
H9B	0.6545	0.8078	0.2938	0.089*	
C10	0.80442 (7)	0.89421 (12)	0.6548 (4)	0.0609 (5)	
C11	0.82924 (9)	0.89602 (15)	0.8595 (4)	0.0796 (6)	
H11	0.8201	0.8570	0.9667	0.096*	
C12	0.86719 (10)	0.95414 (18)	0.9085 (5)	0.0986 (9)	
H12	0.8834	0.9539	1.0473	0.118*	
C13	0.88119 (10)	1.01244 (18)	0.7536 (6)	0.1037 (10)	
H13	0.9069	1.0516	0.7867	0.124*	
C14	0.85711 (10)	1.01259 (15)	0.5506 (5)	0.0936 (8)	
H14	0.8661	1.0524	0.4453	0.112*	
C15	0.81908 (8)	0.95311 (13)	0.5012 (4)	0.0774 (6)	
H15	0.8033	0.9532	0.3615	0.093*	
C16	1.04902 (7)	0.19922 (13)	1.4431 (4)	0.0621 (5)	
C17	1.01297 (9)	0.12496 (14)	1.4512 (5)	0.0835 (7)	
H17A	1.0321	0.0762	1.4999	0.100*	
H17B	0.9839	0.1347	1.5528	0.100*	
C18	0.99320 (11)	0.11388 (12)	1.2193 (4)	0.0875 (7)	
H18A	0.9552	0.1074	1.2189	0.105*	
H18B	1.0089	0.0651	1.1520	0.105*	
C19	1.00905 (7)	0.19155 (10)	1.0914 (4)	0.0585 (5)	
H19	1.0265	0.1758	0.9521	0.070*	
C20	0.96386 (6)	0.25054 (10)	1.0421 (3)	0.0528 (5)	
H20	0.9430	0.2592	1.1775	0.063*	
C21	0.98457 (6)	0.33341 (10)	0.9582 (3)	0.0489 (4)	
H21	1.0036	0.3227	0.8193	0.059*	
C22	1.02418 (7)	0.36991 (10)	1.1231 (4)	0.0573 (5)	
H22A	1.0386	0.4207	1.0623	0.069*	
H22B	1.0061	0.3835	1.2607	0.069*	
C23	1.06905 (7)	0.30999 (11)	1.1726 (4)	0.0644 (5)	
H23A	1.0908	0.3029	1.0411	0.077*	
H23B	1.0910	0.3319	1.2909	0.077*	
C24	0.94118 (7)	0.39470 (10)	0.9057 (3)	0.0503 (5)	
C25	0.94124 (8)	0.43858 (12)	0.7092 (4)	0.0663 (6)	
H25	0.9676	0.4285	0.6051	0.080*	
C26	0.90288 (10)	0.49765 (13)	0.6625 (4)	0.0772 (6)	
H26	0.9039	0.5267	0.5290	0.093*	
C27	0.86400 (9)	0.51280 (13)	0.8116 (4)	0.0760 (7)	
H27	0.8384	0.5525	0.7815	0.091*	
C28	0.86265 (8)	0.46921 (12)	1.0066 (4)	0.0744 (6)	
H28	0.8358	0.4790	1.1089	0.089*	
C29	0.90086 (7)	0.41091 (11)	1.0524 (4)	0.0645 (5)	
H29	0.8993	0.3818	1.1858	0.077*	
N1	0.70898 (6)	0.71810 (10)	0.3111 (3)	0.0613 (4)	
N2	1.04696 (6)	0.23118 (9)	1.2390 (3)	0.0593 (4)	
O1	0.66974 (6)	0.70369 (10)	−0.0274 (2)	0.0818 (4)	
O2	0.82639 (5)	0.71371 (8)	0.6555 (2)	0.0659 (4)	
H2	0.8562	0.7322	0.6361	0.099*	
O3	1.07527 (5)	0.22642 (11)	1.5987 (3)	0.0834 (5)	
O4	0.93197 (5)	0.21245 (8)	0.8770 (3)	0.0692 (4)	
H4	0.9011	0.2126	0.9177	0.104*	

### Atomic displacement parameters (Å^2^)

**Table:**

	*U*^11^	*U*^22^	*U*^33^	*U*^12^	*U*^13^	*U*^23^
C2	0.0443 (10)	0.0671 (12)	0.0678 (13)	0.0000 (9)	0.0060 (11)	0.0075 (12)
C3	0.0637 (13)	0.0894 (15)	0.0783 (16)	0.0137 (12)	0.0105 (13)	−0.0048 (13)
C4	0.0652 (12)	0.0542 (11)	0.1030 (18)	0.0066 (10)	−0.0122 (13)	−0.0077 (13)
C5	0.0479 (10)	0.0502 (10)	0.0753 (13)	0.0015 (8)	−0.0063 (11)	0.0043 (10)
C6	0.0429 (9)	0.0494 (10)	0.0631 (11)	0.0036 (8)	−0.0035 (9)	0.0078 (9)
C7	0.0499 (10)	0.0588 (11)	0.0634 (12)	0.0020 (9)	0.0018 (10)	−0.0035 (10)
C8	0.0584 (11)	0.0573 (11)	0.0930 (16)	0.0153 (10)	−0.0111 (12)	−0.0116 (12)
C9	0.0505 (10)	0.0715 (13)	0.1002 (16)	0.0164 (10)	−0.0136 (12)	−0.0146 (13)
C10	0.0544 (11)	0.0565 (11)	0.0718 (14)	0.0025 (9)	0.0045 (11)	−0.0122 (12)
C11	0.0749 (14)	0.0838 (14)	0.0801 (16)	−0.0052 (13)	−0.0035 (14)	−0.0180 (14)
C12	0.0767 (16)	0.116 (2)	0.103 (2)	−0.0077 (16)	−0.0070 (16)	−0.044 (2)
C13	0.0757 (17)	0.0922 (19)	0.143 (3)	−0.0177 (15)	0.013 (2)	−0.055 (2)
C14	0.0886 (17)	0.0698 (15)	0.122 (2)	−0.0135 (13)	0.0244 (18)	−0.0141 (16)
C15	0.0773 (14)	0.0700 (13)	0.0849 (16)	−0.0060 (12)	0.0042 (13)	−0.0062 (13)
C16	0.0419 (10)	0.0744 (13)	0.0702 (14)	0.0068 (10)	−0.0016 (11)	−0.0008 (12)
C17	0.0671 (13)	0.0843 (15)	0.0992 (18)	−0.0090 (12)	−0.0081 (14)	0.0254 (15)
C18	0.1003 (16)	0.0486 (12)	0.113 (2)	−0.0054 (12)	−0.0228 (17)	0.0072 (13)
C19	0.0606 (11)	0.0459 (10)	0.0689 (12)	−0.0007 (9)	−0.0091 (11)	−0.0050 (10)
C20	0.0477 (9)	0.0468 (9)	0.0640 (12)	−0.0049 (8)	−0.0023 (10)	−0.0097 (9)
C21	0.0482 (9)	0.0446 (9)	0.0539 (10)	−0.0040 (8)	0.0039 (9)	−0.0045 (9)
C22	0.0554 (10)	0.0480 (10)	0.0686 (13)	−0.0098 (9)	−0.0035 (10)	−0.0005 (10)
C23	0.0546 (11)	0.0628 (12)	0.0757 (13)	−0.0126 (10)	−0.0113 (11)	0.0001 (11)
C24	0.0508 (10)	0.0432 (9)	0.0570 (12)	−0.0029 (8)	0.0003 (10)	−0.0059 (9)
C25	0.0712 (12)	0.0676 (12)	0.0600 (13)	0.0037 (11)	0.0034 (11)	0.0009 (11)
C26	0.0881 (15)	0.0690 (13)	0.0744 (15)	0.0075 (13)	−0.0120 (15)	0.0116 (12)
C27	0.0701 (14)	0.0567 (12)	0.1011 (18)	0.0137 (11)	−0.0146 (15)	−0.0048 (14)
C28	0.0708 (13)	0.0628 (12)	0.0896 (16)	0.0149 (11)	0.0144 (12)	−0.0013 (13)
C29	0.0669 (12)	0.0564 (11)	0.0702 (13)	0.0104 (10)	0.0101 (12)	0.0050 (11)
N1	0.0462 (8)	0.0581 (9)	0.0796 (12)	0.0064 (7)	−0.0119 (9)	−0.0075 (9)
N2	0.0567 (9)	0.0544 (9)	0.0668 (11)	−0.0036 (8)	−0.0117 (9)	0.0039 (9)
O1	0.0653 (8)	0.1100 (11)	0.0702 (9)	0.0164 (8)	−0.0082 (8)	0.0074 (9)
O2	0.0508 (6)	0.0617 (8)	0.0853 (9)	−0.0013 (6)	−0.0141 (8)	0.0166 (8)
O3	0.0571 (8)	0.1266 (13)	0.0666 (9)	−0.0098 (9)	−0.0081 (8)	0.0056 (10)
O4	0.0554 (7)	0.0635 (8)	0.0887 (10)	−0.0042 (7)	−0.0151 (8)	−0.0175 (8)

### Geometric parameters (Å, °)

**Table:**

C2—O1	1.228 (2)	C16—N2	1.332 (3)
C2—N1	1.330 (3)	C16—C17	1.509 (3)
C2—C3	1.504 (3)	C17—C18	1.492 (3)
C3—C4	1.488 (3)	C17—H17A	0.9700
C3—H3A	0.9700	C17—H17B	0.9700
C3—H3B	0.9700	C18—C19	1.525 (3)
C4—C5	1.527 (3)	C18—H18A	0.9700
C4—H4A	0.9700	C18—H18B	0.9700
C4—H4B	0.9700	C19—N2	1.456 (2)
C5—N1	1.457 (2)	C19—C20	1.519 (2)
C5—C6	1.521 (2)	C19—H19	0.9800
C5—H5	0.9800	C20—O4	1.420 (2)
C6—O2	1.405 (2)	C20—C21	1.523 (2)
C6—C7	1.534 (2)	C20—H20	0.9800
C6—H6	0.9800	C21—C24	1.513 (2)
C7—C10	1.516 (3)	C21—C22	1.529 (2)
C7—C8	1.519 (3)	C21—H21	0.9800
C7—H7	0.9800	C22—C23	1.523 (2)
C8—C9	1.521 (3)	C22—H22A	0.9700
C8—H8A	0.9700	C22—H22B	0.9700
C8—H8B	0.9700	C23—N2	1.447 (2)
C9—N1	1.452 (2)	C23—H23A	0.9700
C9—H9A	0.9700	C23—H23B	0.9700
C9—H9B	0.9700	C24—C29	1.375 (2)
C10—C15	1.376 (3)	C24—C25	1.377 (3)
C10—C11	1.382 (3)	C25—C26	1.391 (3)
C11—C12	1.376 (3)	C25—H25	0.9300
C11—H11	0.9300	C26—C27	1.354 (3)
C12—C13	1.371 (4)	C26—H26	0.9300
C12—H12	0.9300	C27—C28	1.367 (3)
C13—C14	1.364 (4)	C27—H27	0.9300
C13—H13	0.9300	C28—C29	1.379 (3)
C14—C15	1.393 (3)	C28—H28	0.9300
C14—H14	0.9300	C29—H29	0.9300
C15—H15	0.9300	O2—H2	0.8200
C16—O3	1.229 (2)	O4—H4	0.8200
			
O1—C2—N1	125.73 (19)	C18—C17—H17A	110.6
O1—C2—C3	126.0 (2)	C16—C17—H17A	110.6
N1—C2—C3	108.22 (18)	C18—C17—H17B	110.6
C4—C3—C2	106.58 (18)	C16—C17—H17B	110.6
C4—C3—H3A	110.4	H17A—C17—H17B	108.8
C2—C3—H3A	110.4	C17—C18—C19	106.47 (18)
C4—C3—H3B	110.4	C17—C18—H18A	110.4
C2—C3—H3B	110.4	C19—C18—H18A	110.4
H3A—C3—H3B	108.6	C17—C18—H18B	110.4
C3—C4—C5	106.30 (16)	C19—C18—H18B	110.4
C3—C4—H4A	110.5	H18A—C18—H18B	108.6
C5—C4—H4A	110.5	N2—C19—C20	109.95 (14)
C3—C4—H4B	110.5	N2—C19—C18	103.20 (16)
C5—C4—H4B	110.5	C20—C19—C18	114.52 (17)
H4A—C4—H4B	108.7	N2—C19—H19	109.7
N1—C5—C6	110.71 (14)	C20—C19—H19	109.7
N1—C5—C4	103.92 (17)	C18—C19—H19	109.7
C6—C5—C4	114.52 (15)	O4—C20—C19	107.11 (14)
N1—C5—H5	109.2	O4—C20—C21	110.21 (16)
C6—C5—H5	109.2	C19—C20—C21	110.79 (13)
C4—C5—H5	109.2	O4—C20—H20	109.6
O2—C6—C5	105.47 (13)	C19—C20—H20	109.6
O2—C6—C7	112.52 (16)	C21—C20—H20	109.6
C5—C6—C7	111.74 (13)	C24—C21—C20	113.13 (13)
O2—C6—H6	109.0	C24—C21—C22	111.15 (13)
C5—C6—H6	109.0	C20—C21—C22	110.54 (15)
C7—C6—H6	109.0	C24—C21—H21	107.2
C10—C7—C8	113.73 (16)	C20—C21—H21	107.2
C10—C7—C6	110.46 (13)	C22—C21—H21	107.2
C8—C7—C6	110.69 (16)	C23—C22—C21	111.86 (15)
C10—C7—H7	107.2	C23—C22—H22A	109.2
C8—C7—H7	107.2	C21—C22—H22A	109.2
C6—C7—H7	107.2	C23—C22—H22B	109.2
C7—C8—C9	112.20 (16)	C21—C22—H22B	109.2
C7—C8—H8A	109.2	H22A—C22—H22B	107.9
C9—C8—H8A	109.2	N2—C23—C22	108.88 (14)
C7—C8—H8B	109.2	N2—C23—H23A	109.9
C9—C8—H8B	109.2	C22—C23—H23A	109.9
H8A—C8—H8B	107.9	N2—C23—H23B	109.9
N1—C9—C8	108.03 (14)	C22—C23—H23B	109.9
N1—C9—H9A	110.1	H23A—C23—H23B	108.3
C8—C9—H9A	110.1	C29—C24—C25	116.89 (17)
N1—C9—H9B	110.1	C29—C24—C21	122.11 (17)
C8—C9—H9B	110.1	C25—C24—C21	120.98 (17)
H9A—C9—H9B	108.4	C24—C25—C26	121.7 (2)
C15—C10—C11	117.3 (2)	C24—C25—H25	119.2
C15—C10—C7	122.0 (2)	C26—C25—H25	119.2
C11—C10—C7	120.6 (2)	C27—C26—C25	120.0 (2)
C12—C11—C10	121.6 (3)	C27—C26—H26	120.0
C12—C11—H11	119.2	C25—C26—H26	120.0
C10—C11—H11	119.2	C26—C27—C28	119.5 (2)
C13—C12—C11	120.3 (3)	C26—C27—H27	120.3
C13—C12—H12	119.9	C28—C27—H27	120.3
C11—C12—H12	119.9	C27—C28—C29	120.3 (2)
C14—C13—C12	119.5 (3)	C27—C28—H28	119.8
C14—C13—H13	120.2	C29—C28—H28	119.8
C12—C13—H13	120.2	C24—C29—C28	121.7 (2)
C13—C14—C15	120.0 (3)	C24—C29—H29	119.2
C13—C14—H14	120.0	C28—C29—H29	119.2
C15—C14—H14	120.0	C2—N1—C9	127.00 (18)
C10—C15—C14	121.3 (2)	C2—N1—C5	114.79 (16)
C10—C15—H15	119.3	C9—N1—C5	117.98 (17)
C14—C15—H15	119.3	C16—N2—C23	125.44 (17)
O3—C16—N2	125.70 (19)	C16—N2—C19	114.63 (16)
O3—C16—C17	126.0 (2)	C23—N2—C19	118.29 (16)
N2—C16—C17	108.29 (19)	C6—O2—H2	109.5
C18—C17—C16	105.60 (19)	C20—O4—H4	109.5
			
O1—C2—C3—C4	−177.3 (2)	C19—C20—C21—C24	179.82 (15)
N1—C2—C3—C4	2.8 (2)	O4—C20—C21—C22	−173.17 (13)
C2—C3—C4—C5	−4.3 (2)	C19—C20—C21—C22	−54.8 (2)
C3—C4—C5—N1	4.1 (2)	C24—C21—C22—C23	−178.28 (16)
C3—C4—C5—C6	−116.75 (18)	C20—C21—C22—C23	55.2 (2)
N1—C5—C6—O2	171.79 (16)	C21—C22—C23—N2	−52.5 (2)
C4—C5—C6—O2	−71.1 (2)	C20—C21—C24—C29	49.1 (2)
N1—C5—C6—C7	49.2 (2)	C22—C21—C24—C29	−75.9 (2)
C4—C5—C6—C7	166.31 (17)	C20—C21—C24—C25	−132.62 (18)
O2—C6—C7—C10	63.1 (2)	C22—C21—C24—C25	102.3 (2)
C5—C6—C7—C10	−178.45 (17)	C29—C24—C25—C26	1.1 (3)
O2—C6—C7—C8	−170.01 (15)	C21—C24—C25—C26	−177.25 (17)
C5—C6—C7—C8	−51.6 (2)	C24—C25—C26—C27	−0.5 (3)
C10—C7—C8—C9	−179.74 (18)	C25—C26—C27—C28	−0.5 (3)
C6—C7—C8—C9	55.2 (2)	C26—C27—C28—C29	0.7 (3)
C7—C8—C9—N1	−55.0 (3)	C25—C24—C29—C28	−0.9 (3)
C8—C7—C10—C15	−35.5 (3)	C21—C24—C29—C28	177.43 (18)
C6—C7—C10—C15	89.6 (2)	C27—C28—C29—C24	0.0 (3)
C8—C7—C10—C11	146.7 (2)	O1—C2—N1—C9	−5.6 (3)
C6—C7—C10—C11	−88.1 (2)	C3—C2—N1—C9	174.26 (18)
C15—C10—C11—C12	−0.1 (3)	O1—C2—N1—C5	−179.96 (18)
C7—C10—C11—C12	177.7 (2)	C3—C2—N1—C5	−0.1 (2)
C10—C11—C12—C13	0.3 (3)	C8—C9—N1—C2	−118.1 (2)
C11—C12—C13—C14	0.2 (4)	C8—C9—N1—C5	56.0 (2)
C12—C13—C14—C15	−0.8 (4)	C6—C5—N1—C2	120.80 (18)
C11—C10—C15—C14	−0.5 (3)	C4—C5—N1—C2	−2.6 (2)
C7—C10—C15—C14	−178.30 (18)	C6—C5—N1—C9	−54.1 (2)
C13—C14—C15—C10	1.0 (3)	C4—C5—N1—C9	−177.47 (16)
O3—C16—C17—C18	−176.3 (2)	O3—C16—N2—C23	−9.5 (3)
N2—C16—C17—C18	4.6 (2)	C17—C16—N2—C23	169.56 (17)
C16—C17—C18—C19	−11.2 (2)	O3—C16—N2—C19	−174.60 (17)
C17—C18—C19—N2	13.4 (2)	C17—C16—N2—C19	4.5 (2)
C17—C18—C19—C20	−106.1 (2)	C22—C23—N2—C16	−110.3 (2)
N2—C19—C20—O4	172.89 (15)	C22—C23—N2—C19	54.3 (2)
C18—C19—C20—O4	−71.5 (2)	C20—C19—N2—C16	111.25 (18)
N2—C19—C20—C21	52.6 (2)	C18—C19—N2—C16	−11.3 (2)
C18—C19—C20—C21	168.28 (16)	C20—C19—N2—C23	−55.0 (2)
O4—C20—C21—C24	61.46 (19)	C18—C19—N2—C23	−177.57 (17)

### Hydrogen-bond geometry (Å, °)

**Table:**

*D*—H···*A*	*D*—H	H···*A*	*D*···*A*	*D*—H···*A*
O4—H4···O1^i^	0.82	1.92	2.7366 (19)	175
O2—H2···O3^ii^	0.82	1.88	2.6963 (18)	179

Symmetry codes: (i) −*x*+3/2, *y*−1/2, −*z*+1; (ii) −*x*+2, −*y*+1, *z*−1.
